# Supplementary material for: Exploiting functional regions in the viral RNA genome as druggable entities
Source: eLife. 2025 Jul 2;13:RP103923. doi: 10.7554/eLife.103923 (PMC12221299; doi:10.7554/eLife.103923)
Supplement: Figure 3—figure supplement 7—source data 1. [file elife-103923-fig3-figsupp7-data1.zip › Fig3-figure supplement 7-source data1.pdf]

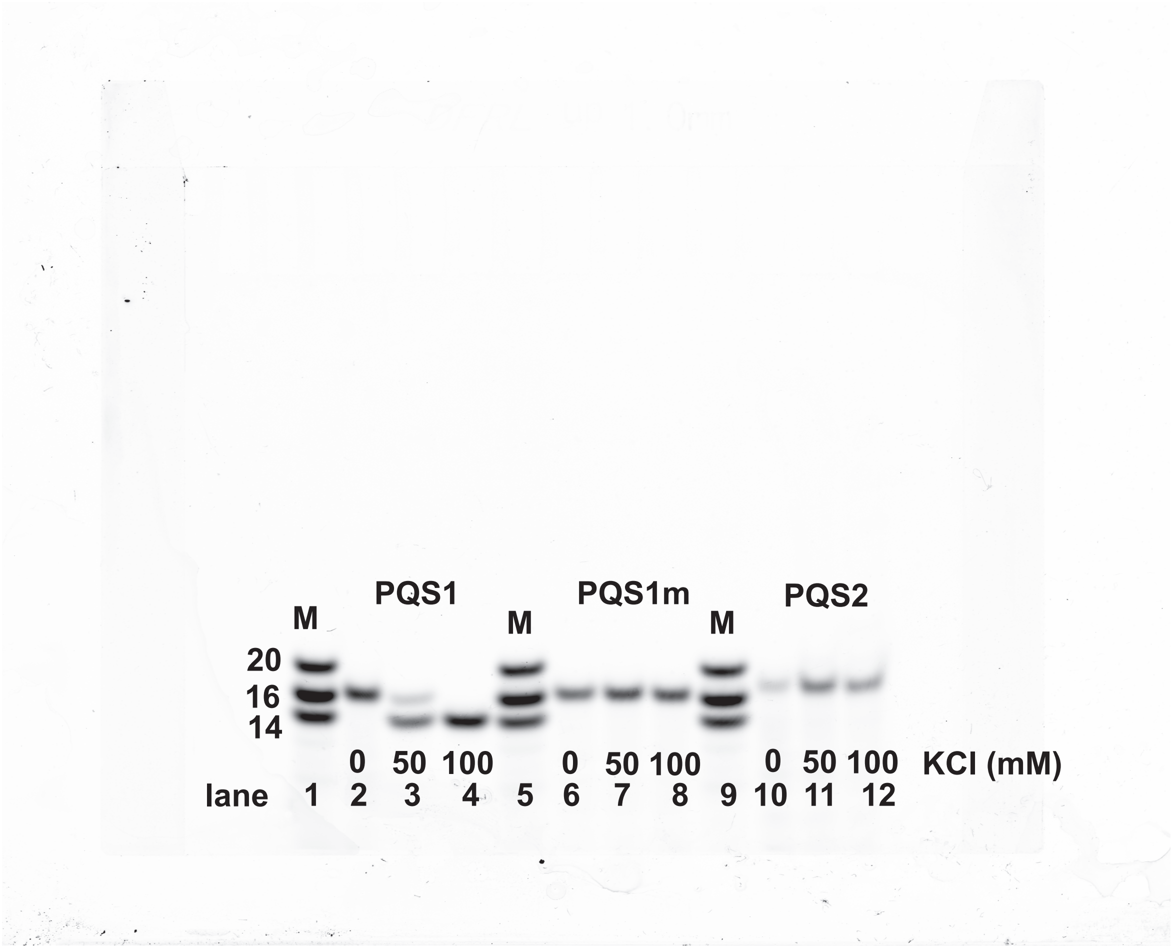

Figure 3—figure supplement 7, source data1. Original gel corresponding to Figure 3B. Native-PAGE analysis of the migration of G4 RNAs (PQS1) and G4 mutant RNAs (PQS1m) was performed under different KCl concentrations. Lanes 1, 5, 9, RNA ladder; lanes 2, 3, 4, PQS1; lanes 6, 7, 8, PQS1m; Lanes 10, 11, 12, PQS2 (not shown in figure 3).
