# Supplementary figures and images for: Exploiting functional regions in the viral RNA genome as druggable entities
Source: eLife. 2025 Jul 2;13:RP103923. doi: 10.7554/eLife.103923 (PMC12221299; doi:10.7554/eLife.103923)

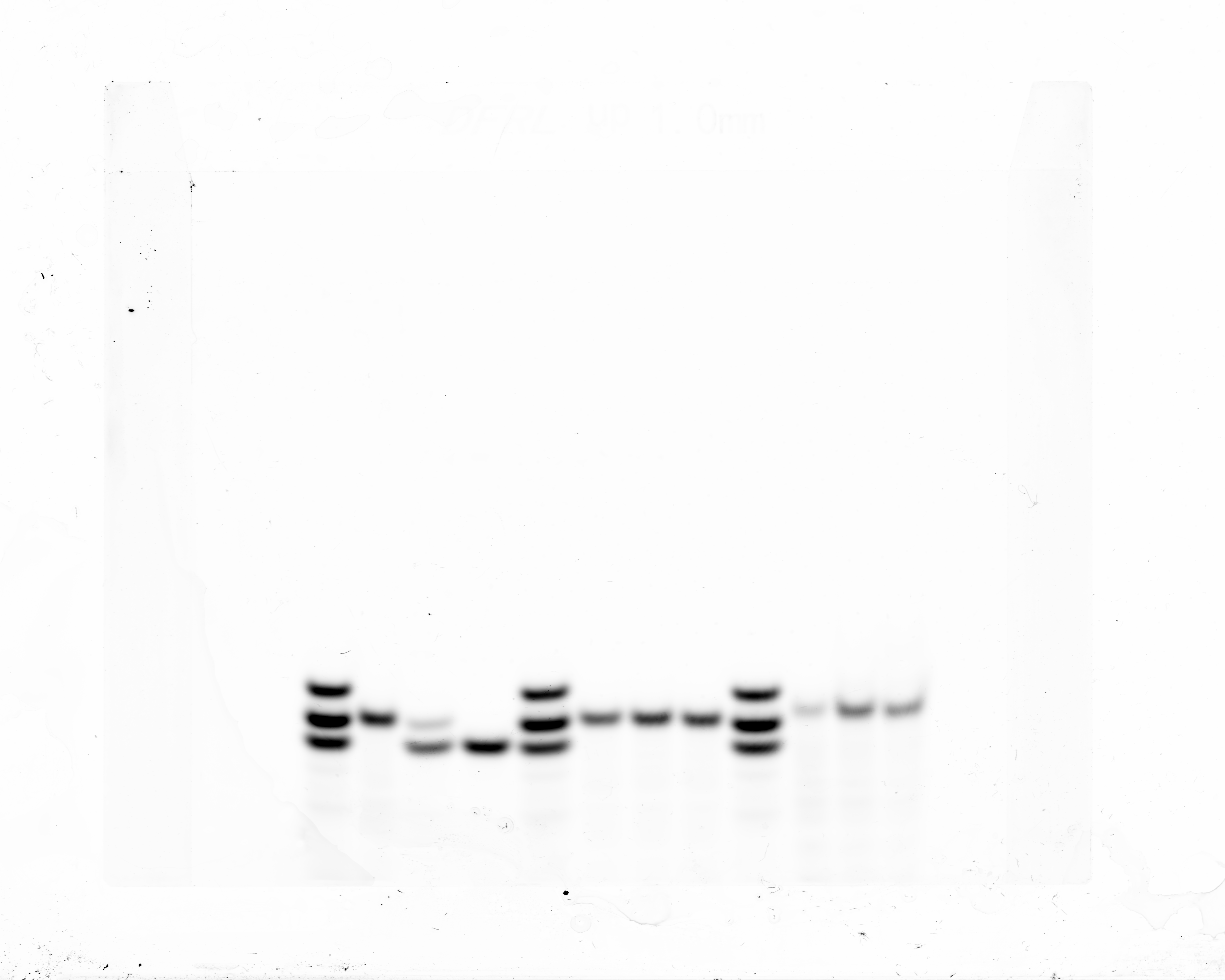

Supplement: Figure 3—figure supplement 7—source data 2. [file elife-103923-fig3-figsupp7-data2.zip › Fig3-figure supplement 7-source data2.tif]

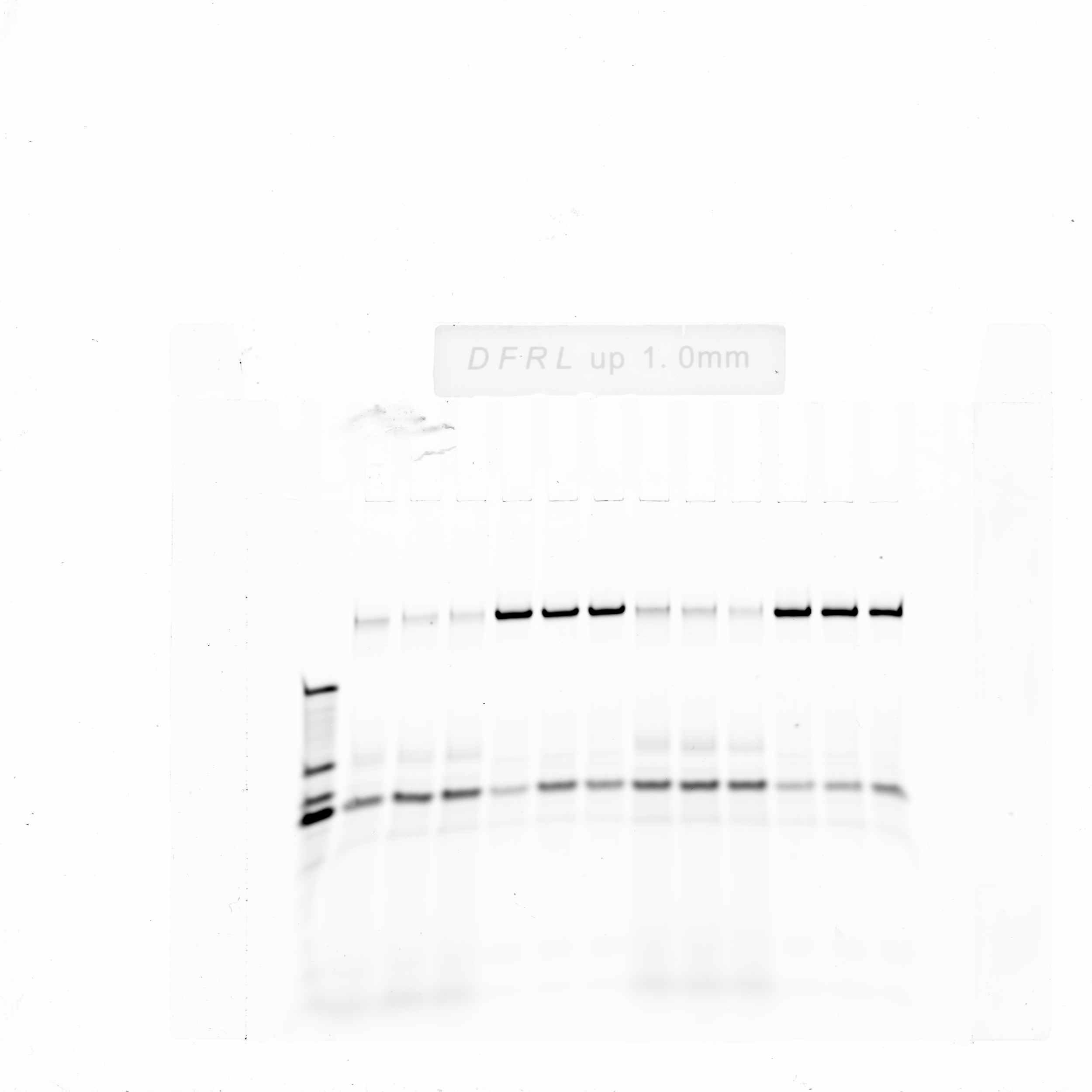

Supplement: Figure 4—source data 2. [file elife-103923-fig4-data2.zip › Figure 4ΓÇöfigure supplement 1-source data2.tif]
