# Supplementary material for: Exploiting functional regions in the viral RNA genome as druggable entities
Source: eLife. 2025 Jul 2;13:RP103923. doi: 10.7554/eLife.103923 (PMC12221299; doi:10.7554/eLife.103923)
Supplement: Figure 4—source data 1. [file elife-103923-fig4-data1.zip › Figure 4ΓÇöfigure supplement 1-source data1.pdf]

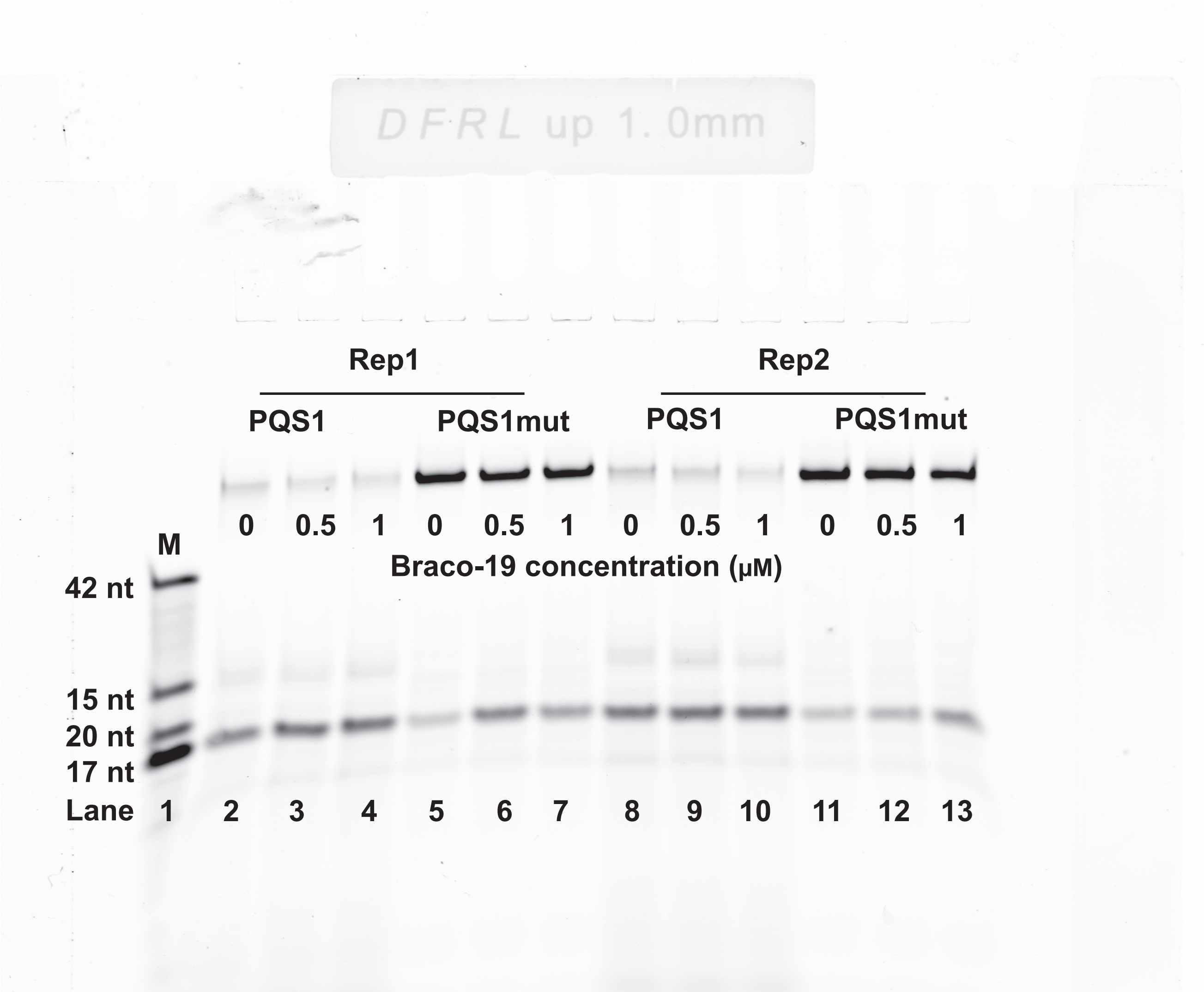

Figure 4—figure supplement 1, source data 1. Original membranes corresponding to Figure 4—figure supplement 1B. Denaturing-PAGE analysis. Black arrows indicate the positions of the full-length product, G4 paused product, and free primer. The fully extended product and the template RNA and the polymerase form a stable complex that moves much more slowly than the ssRNA. Partial full-length extended products and stopped products (due to G-quadruplex fold) were observed along template PQS1 (lane 2), but only fully extended products were observed along G4-mutated template PQS1mut (lane 5). When increasing amounts of compound Braco-19 were incubated with template PQS1, a gradual decrease in fully extended products was observed (lanes 2-4). On the contrary, the fully extended products of template PQS1mut were not affected by the addition of Braco-19, and the G4-specific termination event was not characterized (lanes 5-7). Lane 1, RNA ladder (p15, m17, m20 and m42 in Table S1); lanes 2, no Braco19 control; lanes 3 and 4, 0.5 and 1 μM compounds inhibit RNA extension; lanes 5, no Braco19 control; lanes 6 and 7, compounds do not inhibit RNA extension. Lanes 8-13 are replicate samples of PQS1 and PQS1mut.Lanes 8-13 are replicate samples of PQS1 and PQS1mut.
