# Supplementary material for: Exploiting functional regions in the viral RNA genome as druggable entities
Source: eLife. 2025 Jul 2;13:RP103923. doi: 10.7554/eLife.103923 (PMC12221299; doi:10.7554/eLife.103923)
Supplement: Supplementary file 1. [file elife-103923-supp1.docx]

**Supplementary Table 1. Oligos used in this study.**

| **Oligomer** | **Sequence (5’-3’)** |
| --- | --- |
| PQS1 | GGUUACAGGUGGUUGG |
| PQS1mut | AGUUACAGGUGGUUGG |
| PQS3 | GGCCAAUGGUUCUGGUAAUGG |
| EGFP-PQS1-F | AAGGTTACAGGTGGTTGGGACAGCAAGGGCGAGGAGCT |
| EGFP-PQS1-R | GTCCCAACCACCTGTAACCTTCATGGTGGCGACCGGTAG |
| EGFP-PQS1mut-F | AAAGTTACAGCTGCTTGGGACAGCAAGGGCGAGGAGCTG |
| EGFP-PQS1mut-R | GTCCCAAGCAGCTGTAACTTTCATGGTGGCGACCGGTA |
| PQS1Mut-F1 | CATTTCACAGCACCAAGTTATATGGAGG |
| PQS1Mut-R1 | CCACCTGTAACTTTGATACGATTACCAACA |
| PQS1Mut-F2 | TATCAAAGTTACAGGTGGTTGGGACGATGTTGT |
| PQS1Mut-R2 | ACATCAATGGCCTTTGCTATGCCG |
| PQS3Mut-F1 | ATGGCAAGGTGGTACACGTTA |
| PQS3Mut-R1 | CCATTACCAGAACCATTGGCTAACATCTTA |
| PQS3Mut-F2 | AGCCAATGGTTCTGGTAATGGACAAGCTGT |
| PQS3Mut-R2 | CTTGTTCAAAGGATAACCTGCG |
| Scaffold oligo | AAAAGCACCGACTCGGTGCCACTTTTTCAAGTTGATAACGGACTAGCCTTATTTTAACTTGCTATTTCTAGCTCTAAAAC |
| PQS1m-sgRNA-a | GGATCCTAATACGACTCACTATAGGGACAGCACCAAGTTATATGGGTTTTAGAGCTAGA |
| PQS1m-sgRNA-b | GGATCCTAATACGACTCACTATAGGGATGAGAATTTGTCTCACGGGTTTTAGAGCTAGA |
| PQS3m-sgRNA-a | GGATCCTAATACGACTCACTATAGGGAAGGTGGTACACGTTAAGGGTTTTAGAGCTAGA |
| PQS3m-sgRNA-b | GGATCCTAATACGACTCACTATAGGGCAAACCTTAACAAGAGCGCGTTTTAGAGCTAGA |
| qPCR-nsp12-F | GCTAAGTTGAAGCCAATGCC |
| qPCR-nsp12-R | CCAAATAGTAAAGCCCGCC |
| qPCR-N-F | CCGTGGTGAGCGAATTGAAC |
| qPCR-N-R  β-actin-F  β-actin-R | GGTTCAGTCTTTGCGCCTTC  CCACCATGTACCCTGGCATT  ACTCCTGCTTGCTGATCCAC |
| Template PQS1 | GGUUACAGGUGGUUGG UCGUA UAGUG AGUCG UAUUA |
| Template PQS1mut | AGUUACAGGUGGUUGG UCGUA UAGUG AGUCG UAUUA |
| FAM-P15 | 5’-FAM-UAAUACGACUCACUA-3’ |
| FAM-M17 | 5’-FAM-UAAUACGACUCACUAUA-3’ |
| FAM-M20 | 5’-FAM-UAAUACGACUCACUAUACGA-3’ |
| FAM-M42 | 5’-FAM-UAAUACGACUCACUAUACGAUUAAUACGACUCACUAUACGAU-3’ |
