# Supplementary material for: Exploiting functional regions in the viral RNA genome as druggable entities
Source: eLife. 2025 Jul 2;13:RP103923. doi: 10.7554/eLife.103923 (PMC12221299; doi:10.7554/eLife.103923)
Supplement: Supplementary file 2. [file elife-103923-supp2.docx]

**Supplementary Table 2.** **Characteristics of regions with different SHAPE reactivity and Shannon entropy.**

| Type of the regions | Characteristics | Percentage of the genome | Typical representative regions |
| --- | --- | --- | --- |
| Low SHAPE-low Shannon | Base-paired and stable | 26.40% | 5'UTR, M gene |
| High SHAPE-low Shannon | Accessible and stable | 9.59% | TRS-L, FSE |
| High SHAPE-high Shannon | Accessible and dynamic | 6.23% | E gene |
| Low SHAPE-high Shannon | Base-paired and dynamic | 11.65% | 3'UTR |
