# Supplementary material for: Exploiting functional regions in the viral RNA genome as druggable entities
Source: eLife. 2025 Jul 2;13:RP103923. doi: 10.7554/eLife.103923 (PMC12221299; doi:10.7554/eLife.103923)
Supplement: Supplementary file 4. [file elife-103923-supp4.docx]

**Supplementary Table 4. Binding affinities of Compounds with 5'UTR-SL5 in the PEDV genome.**

| Compounds | Names | structural formula | Binding affinities |
| --- | --- | --- | --- |
| 1 | 1-(2-methylpyrimidin-4-yl)piperidin-4-amine | 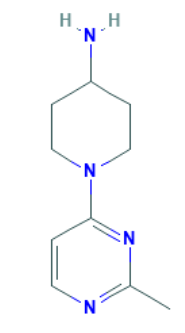 | 4.75 μM |
| 2 | 3,4-Dimethoxybenzylamine | 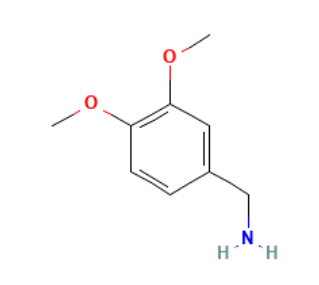 | 0.11 mM |
| 3 | N-ethyl-1-(pyridin-4-yl)piperidin-4-amine | 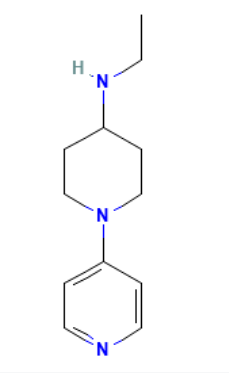 | 18.8 μM |
| 4 | 4-(4-Chlorophenyl)pyrimidin-2-amine | 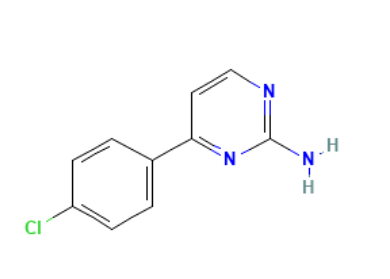 | 1.86 μM |
| 5 | 5-Methoxybenzo[d]thiazol-2-amine | 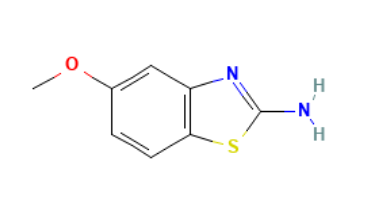 | -- |
| 6 | N-3-pyridinylpyrazolo[1,5-a]pyrimidine-3-carboxamide | 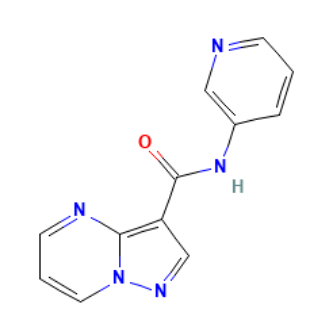 | 1.10 mM |
| 7 | N-methyl-1-(1-phenylpyrazol-4-yl)methanamine | 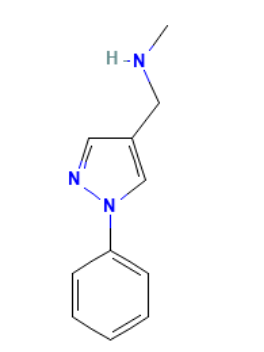 | 13.3 μM |
