# Supplementary material for: Exploiting functional regions in the viral RNA genome as druggable entities
Source: eLife. 2025 Jul 2;13:RP103923. doi: 10.7554/eLife.103923 (PMC12221299; doi:10.7554/eLife.103923)
Supplement: Supplementary file 5. [file elife-103923-supp5.docx]

**Supplementary Table 5.** **PQSs with high conservation in the PEDV genome.**

| number | PQSs | position | length | conservation | location | Characteristics of the region |
| --- | --- | --- | --- | --- | --- | --- |
| PQS1 | GGUUACAGGUGGUUGG | 3109 | 16 | 98.37% | nsp3 | high SHAPE-high Shannon |
| PQS2 | GGUAUUGGUGGUGAGCGG | 10988 | 18 | 97.63% | nsp6 | low SHAPE-low Shannon |
| PQS3 | GGCCAAUGGUUCUGGUAAUGG | 12322 | 21 | 98.52% | nsp10 | low SHAPE-low Shannon |
| PQS4 | GGUUGUUGGCUGGCUAAUGG | 12545 | 20 | 93.79% | nsp10 | low SHAPE-low Shannon |
| PQS5 | GGGCUGGUGGUUUGG | 17720 | 15 | 98.82% | nsp14 | Others |
| PQS6 | GGUGGUAUGGUGCUAGG | 23508 | 17 | 92.16% | S gene | low SHAPE-low Shannon |
| PQS7 | GGCCGUGGUGGGUUUGG | 24607 | 17 | 98.22% | S gene | Others |
