# Supplementary material for: Exploiting functional regions in the viral RNA genome as druggable entities
Source: eLife. 2025 Jul 2;13:RP103923. doi: 10.7554/eLife.103923 (PMC12221299; doi:10.7554/eLife.103923)
Supplement: Supplementary file 6. [file elife-103923-supp6.docx]

**Supplementary Table 6. Genomic locations and structural features of PQS-long chain regions**

| PQS-long chain | Start | End | Length | Structural characteristics |
| --- | --- | --- | --- | --- |
| PQS1 | 3091 | 3276 | 186 nt | high SHAPE-high Shannon |
| PQS2 | 10924 | 11087 | 164 nt | Low SHAPE-low Shannon |
| PQS3 | 12200 | 12590 | 391 nt | Low SHAPE-low Shannon |
| PQS4 | 12336 | 12588 | 253 nt | Low SHAPE-low Shannon |
| PQS6 | 23429 | 23536 | 108 nt | Low SHAPE-low Shannon |
