# Supplementary material for: Exploiting functional regions in the viral RNA genome as druggable entities
Source: eLife. 2025 Jul 2;13:RP103923. doi: 10.7554/eLife.103923 (PMC12221299; doi:10.7554/eLife.103923)
Supplement: Supplementary file 7. [file elife-103923-supp7.docx]

**Supplementary Table 7. Sequence and characterization of target regions of siRNAs.**

| Target region | siRNA | Target Sequences | Local | Conservation | Average SHAPE reactivity | Number of unpaired bases |
| --- | --- | --- | --- | --- | --- | --- |
| Single-Strand | ss-1 | UCAAUUCAACUAAACGAAA | 5'UTR  (58-76 nt) | 88.96% | 0.90 | 15 |
|  | ss-2 | GGCUUAUAAGUCCGUUUUU | Nsp3  (4462-4480 nt) | 97.37% | 0.54 | 10 |
|  | ss-3 | GUACCCUAUUAUUGUUUUC | S  (21738-21756 nt) | 86.90% | 0.81 | 13 |
|  | ss-4 | GGCUGUUUUUAUUUCUCCU | Nsp13  (16899-16917 nt) | 92.25% | 0.44 | 12 |
| Dual-Strand | ds-1 | CCACCAGCACUCAAUGGUA | Nsp2  (2855-2873 nt) | 92.38% | 0.08 | 2 |
|  | ds-2 | GGUGGUGACCAUUACAUCA | ORF3  (25235-25253 nt) | 90.01% | 0.16 | 2 |
|  | ds-3 | UGUUAUUGCCAUCGCUGGC | S  (24128-24146 nt) | 88.96% | 0.09 | 3 |
|  | ds-4 | GAGCGCGAGGCGAUCAUUA | Nsp3  (4613-4631 nt) | 95.40% | 0.27 | 3 |
| Non-target control | siRNA-NC | UCCAGAUCUGUAAGGGUAC | -- | -- | -- | -- |
