# Supplementary material for: Exploiting functional regions in the viral RNA genome as druggable entities
Source: eLife. 2025 Jul 2;13:RP103923. doi: 10.7554/eLife.103923 (PMC12221299; doi:10.7554/eLife.103923)
Supplement: Supplementary file 8. [file elife-103923-supp8.docx]

**Supplementary Table 8. Structural features of anti-SARS-CoV-2 siRNA target regions.**

| siRNA(Bowden-Reid et al., 2023) | Sequence (Sense) | target region | high SHAPE-low Shannon (Manfredonia et al., 2020) |
| --- | --- | --- | --- |
| 2 | 5`-CUUCCCAGGUAACAAACCAdTdT-3` | 5`UTR  (16-34 nt) | Yes |
| 7 | 5`-CGUCCGGGUGUGACCGAAAdTdT-3` | 5`UTR  (241-259 nt) | No |
| 16 | 5`-GUAGUACUUUCUUUUGAACdTdT-3` | Spike  (23093-23111 nt) | No |
| 18 | 5`-GCUACAUCACGAACGCUUUdTdT-3` | Membrane  (27033-27051 nt) | Yes |
| 21 | 5`-GCCAUCCUUACUGCGCUUCdTdT-3` | Envelope  (26338-26356 nt) | No |
| 25 | 5`-GGGUUGCAACUGAGGGAGCdTdT-3` | Nucleocapsid (28668-28696 nt) | Yes |
| 27 | 5`-CGAGAAAACACACGUCCAAdTdT-3` | ORF1ab (NSP1, 292-310 nt) | Yes |
| 30 | 5`-GGCAUUCAGUACGGUCGUAdTdT-3` | ORF1ab (NSP1, 545-563 nt) | Yes |
